# Magnetosomes Extracted from *Magnetospirillum gryphiswaldense* as Theranostic Agents in an Experimental Model of Glioblastoma

**DOI:** 10.1155/2018/2198703

**Published:** 2018-07-11

**Authors:** Silvia Mannucci, Stefano Tambalo, Giamaica Conti, Leonardo Ghin, Alessio Milanese, Anna Carboncino, Elena Nicolato, Maria Rosaria Marinozzi, Donatella Benati, Roberto Bassi, Pasquina Marzola, Andrea Sbarbati

**Affiliations:** ^1^Department of Neuroscience, Biomedicine and Movement Sciences, University of Verona, Strada Le Grazie 8, 37134 Verona, Italy; ^2^Consorzio INSTM, Via G. Giusti 9, 50121 Firenze, Italy; ^3^Department of Biotechnology, University of Verona, Strada Le Grazie 15, 37134 Verona, Italy; ^4^Department of Computer Science, University of Verona, Strada Le Grazie 15, 37134 Verona, Italy

## Abstract

Magnetic fluid hyperthermia (MFH) with chemically synthesized nanoparticles is currently used in clinical trials as it destroys tumor cells with an extremely localized deposition of thermal energy. In this paper, we investigated an MFH protocol based on magnetic nanoparticles naturally produced by magnetotactic bacteria: magnetosomes. The efficacy of such protocol is tested in a xenograft model of glioblastoma. Mice receive a single intratumoral injection of magnetosomes, and they are exposed three times in a week to an alternating magnetic field with concurrent temperature measurements. MRI is used to visualize the nanoparticles and to monitor tumor size before and after the treatment. Statistically significant inhibition of the tumor growth is detected in subjects exposed to the alternating magnetic field compared to control groups. Moreover, thanks to magnetosomes high transversal relaxivity, their effective delivery to the tumor tissue is monitored by MRI. It is apparent that the efficacy of this protocol is limited by inhomogeneous delivery of magnetosomes to tumor tissue. These results suggest that naturally synthesized magnetosomes could be effectively considered as theranostic agent candidates for hyperthermia based on iron oxide nanoparticles.

## 1. Introduction

Glioblastoma multiforme is the most frequent and malignant type of glioma, with a mean survival of fewer than 15 months despite the advances in diagnoses and treatments now available [1, 2]. Novel therapeutic agents and approaches to increase the survival rates, as well as clinical protocols to eventually cure this type of disease, are thus highly needed.

In oncology, the term hyperthermia refers to the treatment of malignant diseases by administering heat in various ways. The process of rising body temperature, either locally or globally for medical purposes, is usually applied as an adjunct to an already established treatment modality (radiotherapy and chemotherapy). Temperatures in the range of 40–46°C are needed to trigger cancer cell apoptosis by temperature-driven alterations in molecular pathways. At the same time, local temperatures should be maintained under 44°C to avoid damage to tissues surrounding the target, and the whole body temperature should remain under 42°C, which is the upper limit compatible with life [3, 4]. Conventional thermotherapy procedures use different energy sources for the generation of heat within the tissue: radiofrequency waves, ultrasound, and electric or magnetic fields [5, 6]. The cellular effect of thermal energy deposition is very complicated. Briefly, hyperthermia may kill or weaken tumor cells and is controlled to limit effects on healthy cells. Tumor tissues are characterized by a disorganized and compact vascular structure which negatively impacts on tissue perfusion, thus reducing their capabilities to efficiently dissipate heat induced by energy transfer. It is proven that intense heating will cause denaturation of proteins and other biochemical consequences (slowed cell division, altered molecular pathways, and inhibited synthesis of proteins) that ultimately lead to cell apoptosis [7]. On the contrary, healthy tissues around the tumor can more easily maintain the temperature in the physiological range and compensate for mild heating stress, minimizing the effects of hyperthermia [3].

Within this frame, magnetic fluid hyperthermia (MFH) represents a further development in the area [8] and is currently under testing in some preclinical studies [9] and clinical trials [10]. This method delivers thermal energy to the target region by superparamagnetic iron oxide nanoparticles exposed to an alternating magnetic field (AMF): when magnetic nanoparticles (MNPs) are injected in the tumor and an AMF is applied, the tumor temperature rises and results in extremely selective thermal ablation of tumor cells [11].

MNPs such as magnetite, hematite, and maghemite, which are mainly obtained by chemical synthesis, are the most frequently investigated nanoparticles for biomedical applications because of their biocompatibility [12]. Recently, researchers have devoted their attention to a class of iron oxide MNPs naturally produced by magnetotactic bacteria, named magnetosomes (MNs) [13–15]. Magnetosomes are widely described in the literature and well characterized in terms of magnetic properties, interactions with living tissues, and thermal efficiency [16–18]. MNs are characterized by a very high specific absorption rate (SAR), a measurement of their efficiency in transforming the energy of the applied magnetic field into heat, and high T2 relaxivity, relevant for their visualization in MRI [17]. Alphandery et al. [16] have applied chains of magnetosomes extracted from *Magnetospirillum magneticum* to treat experimental models of breast cancer. Previous work from our group [19] suggested a possible application of MFH protocols that make use of MNs extracted from *Magnetospirillum gryphiswaldense*. Magnetosomes extracted from *Magnetospirillum gryphiswaldense* have been characterized as potential contrast agents for MRI [17] by measuring their longitudinal and transversal relaxivity: they have been recognized as effective contrast agents for MRI since they are characterized by *r*_2_ ≈ 200 mM^−1^·s^−1^ at 4.7 T [18]. In this paper, we report the application of chains of magnetosomes extracted from *Magnetospirillum gryphiswaldense* in an experimental model of glioma with specific attention to their theranostic properties, that is, the capability to act as contrast agents in MRI and as agents for magnetic fluid hyperthermia. This work aims at further investigating the biocompatibility of MNs on cancer cell cultures and quantitatively evaluating the outcome of intratumoral heating therapy in a xenograft murine model of glioblastoma. The MFH protocol presented here was designed paying serious attention towards the translational value of the method, putting adequate resting periods in between exposures and adopting levels of thermal energy deposition constrained by biosafety limits for human trials.

## 2. Materials and Methods

### 2.1. Production and Extraction of MNs

Magnetosomes were extracted from *Magnetospirillum gryphiswaldense* MSR-1 culture and purified according to the protocol proposed by Grünberg et al. [19] and detailed in Mannucci et al. [17] After purification, MNs were dried for 5 h using a lyophilizer, irradiated with *γ* rays (56 Gy for 84 min) and finally stored at −20°C. For their use in in vitro/in vivo experiments, MNs were dispersed in PBS and sonicated until the suspensions appeared homogeneous and transparent at visual inspection.

### 2.2. In Vitro Experiments and Cell Cultures

Human glioblastoma-astrocytoma, epithelial-like cells (U87MG, purchased by ATCC Manassas, VA), were cultured as detailed in Supplementary Information S1.

The internalization of MNs into U87MG cells was assessed by optical microscopy. After incubation with MNs (experimental details reported in Supplementary Information S2), cells were double stained with Prussian blue, to visualize iron, and Nuclear Fast Red (Bioptica, Italy), to visualize cell nuclei. U87MG cells were observed at 10x, 20x, and 40x magnification using an optical Olympus microscope (BX-URA2, Olympus optical, GMBH, Hamburg, Germany) equipped with Image ProPlus software (Media Cybernetics, Rockville, USA). For transmission electron microscopy (TEM), U87MG cancer cells were plated on a 2.4 cm culture glass, stained with lead citrate, and observed using Philips Morgagni TEM and equipped with a Megaview II camera for digital image acquisition as described in Supplementary Information S3.

Cell viability (CV) was evaluated by the 3-(4,5-dimethylthiazol-2-yl)-2,5-diphenyltetrazolium bromide (MTT) test. Cells were plated at a density of 5000 cells per well in 96-well plates and incubated at 37°C in a mixture of air and 5% CO_2_. After 24 h, the medium was replaced with fresh medium containing 1, 0.5, and 0.2 mg/ml of sterilized MNs, respectively. After 6, 12, and 24 h of incubation, 100 *µ*l of MTT (at 5 mg/ml concentration, purchased from Sigma, Italy) was added to each well and incubated for additional 4 h (37°C, 5% CO_2_). Afterward, plates were removed from the incubator, and formazan crystals, which originate from plasma membrane electron transport in viable cells, were dissolved in 100 *µ*l of DMSO (Sigma, Italy). The multiwell was placed into a monochromator (ChroMate Awareness Technology) for the measurement of absorbance at 570 and 630 nm. Four measurements of optical density (OD) were recorded for each sample, and cell viability (%) was calculated with the following equation: CV% = (OD_sample_/OD_control_) × 100.

### 2.3. In Vivo Experiments: Experimental Design and Thermotherapy Protocol

**Figure 1:**
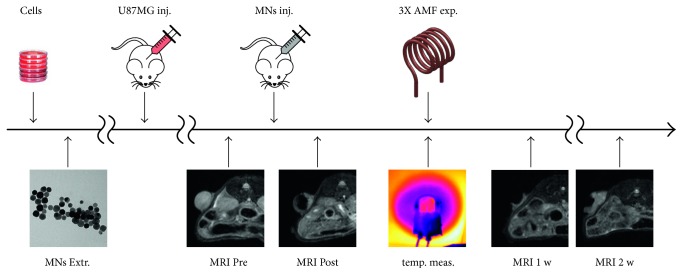
Experimental protocol timeline. Magnetosomes were extracted and injected in tumors under MRI monitoring; same scans were used to measure the pretreatment size of the tumor. Thermal maps and local temperature variations were measured during hyperthermic treatment. Upon completion of thermotherapy, immediately prior to histology, a new MRI session was conducted to measure the final volume of tumors.

Thirty-two nude homozygote male mice (Harlan Laboratories, Udine, Italy) were maintained under standard environmental conditions (temperature, humidity, and 12 h/12 h light/dark cycle, with water and food ad libitum) under veterinarian control in the animal facility of the University of Verona. The experimental plan received authorization from the Italian Ministry of Health (approval numbers 27/2012-B) and was approved by the Animal Care and Use Committee of the University of Verona. Animal work was conducted following Italian law (D.L. 4 March 2014 no. 26) and the European Union normative (2010/63/EU). Major efforts were performed to minimize the number of animals and to avoid their suffering. A graphical illustration of the experimental protocol timeline is reported in Figure 1. One million U87MG cells resuspended in 200 *µ*l of sterile PBS were subcutaneously injected in the right flank of anesthetized mice. The size of the tumor mass was measured every other day with a hand-held caliper, and the gross volume was calculated as (*D* ∗ d^2^)/2, [20] where *D* = maximum diameter and *d* = minimum diameter of the mass. When tumor volume reached 100 *µ*l (30 ± 5 days after cells injection), magnetic resonance imaging (MRI) was performed to check their effective volume. At this point, mice were randomly divided in three groups: (i) *n*=10, animals received intratumorally 100 *µ*l of saline (Group S); (ii) *n*=10, animals received 1.5 mg of MNs diluted in 100 *µ*l of PBS using an intradermal needle (27G) (Group M); (iii) *n*=12, animals received 1.5 mg of MNs diluted in 100 *µ*l of PBS (Group HT) and were exposed to AMF every other day for one week after injection of MNs. The AMF apparatus (Magnetherm, nanoTherics UK) yields a magnetic field intensity of 23 kA/m (≈29 mT) and frequency of 110 kHz. These values are very close to those used in human application of hyperthermia [8]. Mice were placed on a custom animal holder under isofluorane anesthesia 1.5% (Forane, Abbott) and exposed to AMF for 20 min. A digital IR camera (Flir I7, Flir Systems, Italy) was used to take snapshots of heating distribution in the tumor and surrounding tissues. In addition to the thermal camera, a multichannel thermometer equipped with optical fiber probes (FOTEMP4, Optocon AG, Germany) was used to assess temperature variation within the tumor and the body. Under anesthesia, probes were placed by a nonmetallic catheter inside the tumor mass and under the skin of the contralateral flank of the mouse; temperature was continuously recorded during AMF exposures. All mice were monitored by MRI before, 24 h, one week and two weeks after the intratumor injection of MNs (or saline). The first time point was acquired to measure the initial tumor volume; the second time point was chosen to evaluate the intratumor distribution of MNs, and the last time points served to monitor the efficacy of MFH in reducing tumor mass. Following the last MRI scan, mice were sacrificed by isofluorane overdose and by neck dislocation; the tumors were then excised for histological analysis. Animals were placed prone in a heated MRI animal holder, and a 3.5 cm i.d. transmitter/receiver birdcage coil was used. T_2_- and T_2_^*∗*^-weighted images were acquired to detect the tumor and the presence of MNs, respectively. MR imaging details are reported in Supplementary Information S4.

To measure the volume of tumors, MR images were processed with custom software coded in MATLAB (MathWorks, Natick, MA). The tumor area was manually selected slice by slice by entering a series of points and extracting the polygon represented by this set of points. The polygon area was then segmented into three parts: the total tumor surface, the region occupied by magnetosomes, and their difference, which corresponds to the area of the tumor that is free of magnetosomes. The portion of the polygon that contains MNs was then extracted via Otsu's thresholding. The percentual increase in tumor volume was calculated at each time point as 100 ∗ (*V*(*t*)/*V*(0)), where *V*(*t*) is the total tumor volume at time *t* and *V*(0) is the initial volume.

### 2.4. Histology

After the last MRI acquisition, animals were sacrificed. Tumors were excised, washed, and embedded in paraffin, and details on the procedure are available in Supplementary Information S5. Sections were ultimately stained with Prussian blue and Nuclear Fast Red (Bioptica, Italy) to visualize iron nanoparticles and nuclei, respectively.

## 3. Results

### 3.1. In Vitro Experiments

**Figure 2:**
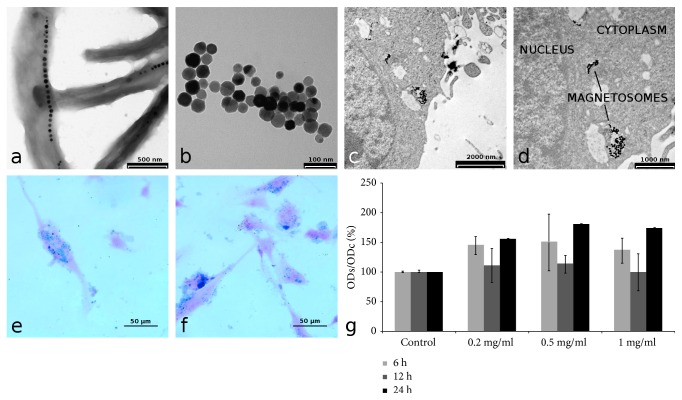
In vitro characterization of MNs. (a) TEM of whole-mount of bacteria (scale bar, 500 nm). (b) MNs after isolation of *M. gryphiswaldense* (scale bar, 100 nm). (c, d) TEM of the internalization of MNs with U87MG cells: MNs are visible in cytoplasm vacuoles (scale bar, (c) 2000 nm and (d) 1000 nm). (e, f) Cellular uptake: representative histological images of MNs-treated cells after 24 h of incubation with 0.2 and 0.5 mg/ml of MNs (Prussian blue staining; scale bar, 50 *µ*m). (g) MTT assay on U87MG cells after incubation with MNs: MTT assay shows a negligible cytotoxic effect of MNs, percent viability of cells incubated with MNs is expressed relative to control cells. Error bars refer to SEM.

TEM images of magnetotactic bacteria observed by a backscattered electron detector clearly show the organization of MNs in chains along the longitudinal axis of bacteria (Figure 2) and the cuboctahedral structure of single nanoparticles (Figure 2), as reported by Mannucci et al. [17]. The MNs preparations used in the present work were previously characterized for chemical and physical properties [18]. Briefly, the nanocrystal size is 42 ± 9 nm, and MNs show a superparamagnetic behaviour and are characterized by transversal relaxivity comparable to that of commercial superparamagnetic contrast agents. Importantly, the presence of a phospholipid membrane provides a good protection against oxidation so that the MNs oxidation state is stable over months. The specific absorption rate (SAR) was also measured by applying an alternate magnetic field of 17 kA/m and 183 kHz and resulted in 482.7 ± 50.8 W/g per mass of iron, a value that is among the highest reported in the literature.

The uptake of MNs by U87MG cancer cells was investigated by TEM and Prussian blue staining of U87MG cells after incubation with MNs; the effect of varying MNs concentration in the culture medium and incubation time was investigated. Qualitative analysis of Prussian blue stained samples showed that the best condition for internalization was 0.2 mg/ml of purified and sterilized MNs for an incubation time of 24 h. TEM was used to investigate in deep the cellular sites of MNs uptake. As a general finding, TEM images revealed that MNs are distributed on the cellular membrane, within the cytoplasm and near the nucleus (Figures 2 and 2). MTT assay revealed negligible cellular toxicity of sterilized MNs. Results of toxicity tests are reported in Figure 2: different MNs amounts (1 mg/ml, 0.5 mg/ml, and 0.2 mg/ml) incubated for different time periods (6, 12, and 24 h) showed no statistically significant effect on cells viability. It could be surprising that an increase in optical absorbance was detected after 24 h of incubation with MNs that could be interpreted as an increase in cell viability or proliferation induced by MNs. However, such an increase was not statistically significant.

### 3.2. In Vivo Experiments

**Figure 3:**
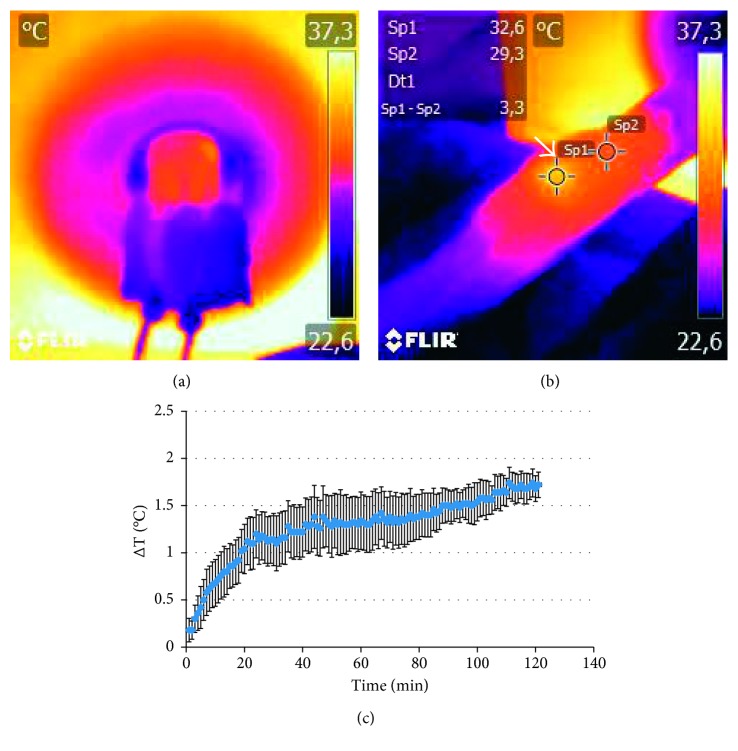
Analysis of temperature within tumor mass. (a) Thermal map acquired with the animal placed inside the coil. (b) Two ROIs placed on the thermal maps of the body and tumor immediately after the treatment. (c) Difference between the tumor and body temperature measured by optical fiber probes as a function of exposure time; data are reported as average ± SEM. Difference between the body and tumor become statistically significant after about 8 min of exposure.

Temperature variations in animals treated with AMF were measured during the exposure both with thermal IR camera and optical fiber thermometer. Figures 3(a) and 3(b) report representative thermal maps acquired during (a) or immediately after (b) application of AMF in one mouse belonging to the HT group; the higher temperature of the tumor compared to the body is visible on the right flank as indicated by the arrow (Figure 3(b)). We should mention that it was not possible to record thermal maps for all subjects: some major issues such as anatomical localization of the tumor and subsequent positioning of the animal inside the coil did prevent the tumor mass to be visible through the viewfinder of the camera. Optical fiber probes were used instead of the sample body and tumor temperature in each subject every 10 s for the whole exposure time and recorded for later processing.  Figure 3(c) shows the difference of temperature ΔT (mean ± SEM) between the tumor and body. Heating of the tumor mass was recorded, and the difference with the measured body temperature became statistically significant after about 8 min of exposure, as reported in Figure 3(c).

**Figure 4:**
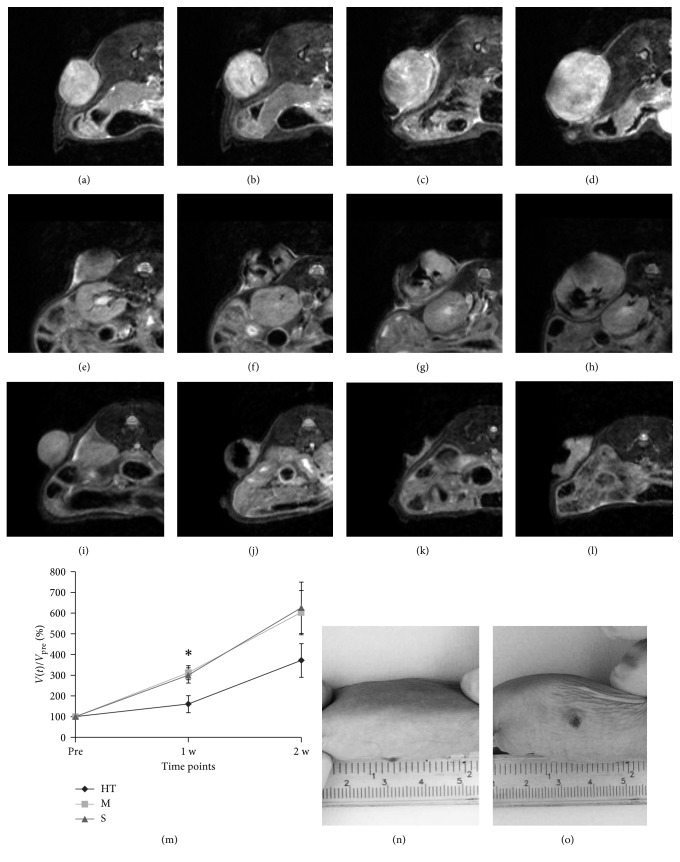
MR images of representative animals. (a–d) Animal treated with saline injection; images acquired before saline injection (a), 24 h (b), one week (c), and two weeks (d) after saline injection. (e–h) Animal treated with MNs injection; images acquired before MNs injection (e), 24 h (f), one week (g), and two weeks (h) after MNs injection. (i–l) Animal treated with MNs injection with AMF; images acquired before MNs injection (i), 24 h (j), one week (k), and two weeks (l) after MNs injection. (m) shows the relative tumor size for the three experimental groups as a function of time (^*∗*^ *p* < 0.05). (n) and (o) show two pictures of a representative animal treated with AMF and MNs in which we obtained good response to thermotherapy.

**Figure 5:**
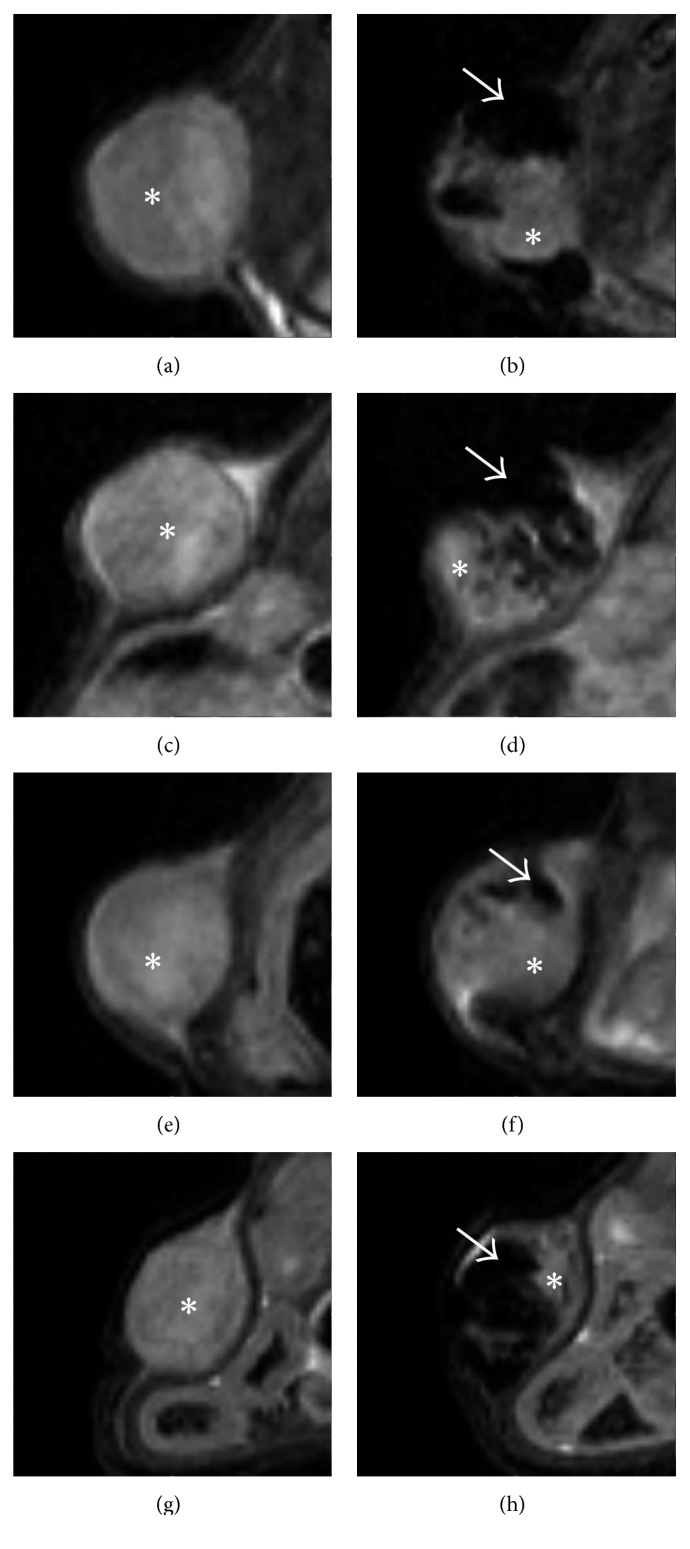
Intratumor injection produces highly inhomogeneous distribution of MNs within tumor tissue. Representative images of tumors acquired before (a, c, e, g) and 24 h after (b, d, f, h) injection of MNs. Within the tumor region, there are areas almost completely free of MNs (asterisks) and regions with high MNs content (arrows).

Volumetric T_2_w MR images were obtained for each subject both for experimental and control groups. A first set of images was necessary to assess the initial volume of the tumor; these were lately used as reference to calculate the percentage increase in tumor volume. Immediately after the administration of MNs, but prior to hyperthermia treatment, a second set of images was acquired to localize MNs within the tumor masses. An additional set of images was acquired immediately after the treatment (1 w) and one week later (2 w) to evaluate efficacy of the therapy and to estimate the final volume of tumors. For each experimental group, representative MR images of the development of U87MG tumors are reported in Figure 4. Figures 4(a)–4(d) report the growth of tumors for Group S, administered with saline only. At 2 w time point, the MR signal of the mass is inhomogeneous, with signs of tissue degeneration marked by areas of moderate hyperintensity. Figures 4(e)–4(h) show the progression of the tumor in Group M. These mice did not receive any MFH treatment. MNs inside the tumor mass are clearly detectable as dark areas. Figures 4(i)–4(l) show the tumor evolution in one animal treated with MFH. One week after injection, the almost complete remission of the tumor after MFH treatment, with only a thin layer of neoplastic tissue, was visible. The effect was also evident in the picture of this subject at this time point (Figures 4(n) and 4(o)), where necrotic tissue is well visible at the tumor site. This effect was detected for two subjects in Group HT, while the remaining showed only minor signs of tissue damage at the macroscopic level. From Figure 4(j), it is clearly evident that, 24 h after administration, there is a fraction of the tumor volume completely free from nanoparticles since it appears hyperintense in T_2_w images with signal intensity similar to the preinjection value (Figure 4(i)). In the subsequent images (Figures 4(j) and 4(k)), the fraction of the tumor volume that was initially free from magnetosomes keeps growing while MFH efficacy is limited to the central region of the tumor where magnetosomes were efficiently delivered. In order to better show how intratumoral injection produces inhomogeneous distribution of MNs within tumor tissue, in Figure 5 we have reported with high magnification representative MR images of tumors acquired prior to and 24 h after administration of MNs in four representative mice. By comparing pre- and post-treatment images, it can be clearly seen that within the tumor region, there are areas almost completely free of MNs (showing hyperintense signal equal to pretreatment) and regions with high MNs content (showing hypointense signal at the level of background noise). These findings indicate that efficient and homogeneous intratumoral delivery of magnetosomes is crucial and that their diagnostic property, namely, the capability to substantially affect MRI signal intensity, is of paramount importance in future clinical applications. Relative tumor size for each experimental group, as a function of time, is reported in Figure 4(m). Values are expressed as percentage of the initial size, measured before administration of MNs or saline, for each subject. Data were reported as mean ± SEM. At the first time point, upon completion of MFH therapy, the size of Group HT is reduced when compared to control groups (relative tumor size: 161 ± 23%, 314 ± 33%, and 300 ± 36% for Groups HT, M, and S, resp.). One week later, at time point 2 w, the relative tumor size in the HT group remains smaller than in the remaining groups (371 ± 58% (HT), 602 ± 106% (M), and 626 ± 124% (S)). Statistical significance was tested among the three groups by one way ANOVA: relative tumor size for Group HT is significantly lower than tumor size for both Groups M and S at 1 w (*p* < 0.01), while the difference was not statistically significant between the two controls.

### 3.3. Histology

**Figure 6:**
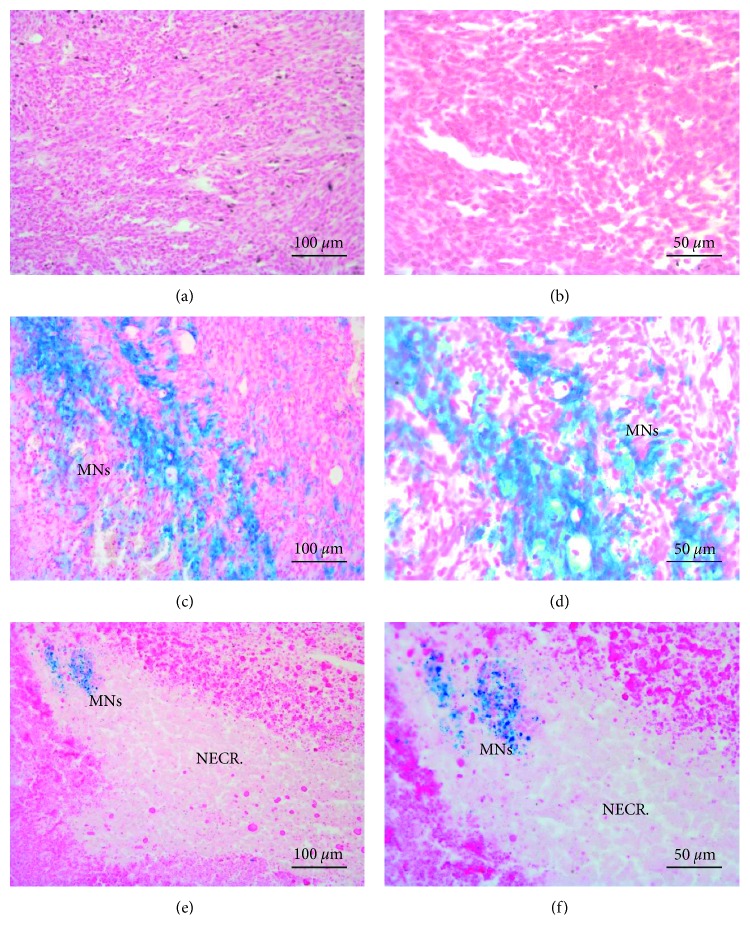
Histological analysis of tumors. (a, b) Histological sections of tumors treated with saline. Morphology of cancer cells is preserved with negligible signs of tissue modifications. (c, d) In tumors treated only with MNs, nanoparticles are clearly marked by Prussian blue staining. No evident signs of cytotoxic effects were detectable. (e, f) Tumors injected with MNs and exposed to AMF showed clusters of MNs surrounded by large areas of tissue necrosis induced by hyperthermic treatment (scale bar, 100 *µ*m and 50 *µ*m for the left and right column, resp.).

Tumors treated with sterile saline solution (Group S, Figures 6(a) and 6(b)) exhibit little to no modifications in the parenchyma with a good preservation of cellular morphology. Spontaneous necrosis occurred in the deeper portion of the mass due to a reduction of capillary density in those areas. Histological slices of tumors belonging to Groups HT and M showed the presence of MNs, clearly identified by blue staining. In particular, slices of tumors treated with MNs but not exposed to AMF (Group M, Figures 6(c) and 6(d)) showed no evident signs of cytotoxic effects in regions were large amounts of Fe were detected by Prussian blue staining. Moreover, the nanoparticles were clustered closer to the injection's sites and were detectable also in tumor parenchyma, even if in lower concentrations. In tumors treated with MNs and exposed to AMF (Figures 6(e) and 6(f)), large necrotic areas characterized by a paucity of cells and the presence of thin collagen fibers that substitute dead tissue were visible in different portions of tumor slices. Moreover, the presence of clustered MNs within the edges of necrotic lesions could be an evidence of the effectiveness of hyperthermic treatment associated with the administration of MNs. Figures 6(e) and 6(f) also show that necrotic areas have reduced blue staining probably due to lymphatic drainage of MNs.

## 4. Discussion

Iron oxide magnetic nanoparticles are known to generate heat because of molecular vibration in the presence of AMF [9]. MFH is a fairly new concept that finds its applications in the treatment of different types of cancers previously loaded with MNPs and exposed to an AMF. The key mechanism of this treatment is based on the intracellular heat stress resulting in activation of several cellular degradation processes (protein denaturation, reduced perfusion, and changes in pH, among others), which ultimately lead to apoptosis [21, 22]. Magnetosomes are proven to be equally effective both for therapy, as heating mediators, and for MR imaging, as contrast agents [23]: these properties can qualify this class of naturally synthesized nanoparticles as theranostic agents. In previous work, we tested the theranostic properties of MNs on a xenograft colon cancer model [17]; here, we have further developed our protocol for MFH and demonstrated *in vivo* the substantial inhibition of the tumor growth in a murine model of glioblastoma multiforme. The proposed method is minimally invasive, and a standard MRI session can provide evidence on the efficacy of the treatment, progression of the disease, and tracking of the antitumoral magnetic agents at the same time: a convenient feature towards future clinical applications.

Our MRI images, acquired at different time points during cancer progression, have demonstrated that, after intratumoral injection, MNs remained within the tumor for the whole period of observation with no evidence of migration to other organs or tissues, as also confirmed by other groups [16, 24]. This feature allows for repeated and highly specific AMF treatments after a single administration of the magnetic material, minimizing unwanted side effects (e.g., heating of body regions not affected by the disease and thus not involved in the treatment plan). The distribution of MNs strongly depends on the route of administration. Today, one of the most critical challenges in hyperthermia is the accumulation of magnetic agents in the tumor after systemic administration [25, 26]. Their removal from the blood stream, the development of methods to increase specific tissue uptake, and consequently avoiding unwanted accumulation in healthy tissues are currently among the major unresolved issues in the field. Until these challenges are resolved, intratumoral injection remains the most effective technique to load a sufficient amount of the magnetic material within a neoplastic mass for therapeutic purposes [26, 27]. Thanks to high transversal relaxivity, effective and homogeneous delivery of MNs to tumor tissue by intratumoral injection can be monitored by MRI and possibly improved before MFH treatment. Thermal maps of our animals exposed to AMF and treated with MNs show a well-defined area of increased temperature corresponding to the whole volume of the tumor. At the same time, the body temperature sampled from the contralateral flank of the subject remains constant for the entire duration of the AMF application. The homogeneous and selective heating, measured on the surface of the implanted glioblastoma and confirmed by optical temperature probes, can be explained by the presence of well-localized clusters of MNs, a hypothesis confirmed by MRI and histology. These heating spots can generate intratumoral temperature gradients that might lead to cancer cell death within a certain distance from the MNs [24], an effect that could partially compensate the uneven distribution of nanoparticles in the tumor.

The temperature increase produced by MNs in the tumors of our experiments was lower than values commonly reported for MFH [28, 29]. However, it has been recently reported that local heating decays exponentially with increasing distance from the nanoparticle surface over a few nanometers [30] so a moderate increase in the temperature, as measured by optical fiber probes, may indeed correspond to substantially higher increases at the nanoparticle surface. Concerning approaches for tumor regression, similar findings were described by other groups, and some evidence is supporting the hypothesis that less aggressive therapies could produce results comparable to more radical treatments [24, 31]. The moderate—yet highly effective—heating we achieved during AMF exposure could be explained by the interaction of MNs with the surrounding environment. Despite the elevate SAR value measured for lyophilized samples of MNs [18], their heating efficiency could be reduced when they are included in a living tissue [32, 33]. Biological structures as cellular membranes and vesicles could lead to immobilization of the nanoparticles and minimization of Brownian motion with subsequent partial restriction of heating mechanisms [34]. Being aware of these potential limitations, we found that heating power emanated by MNs induced a marked alteration in tumors that received MFH treatment; such effect ultimately resulted in a significant reduction of tumor size after the therapy. On the contrary, in the absence of AMF, MNs are inert: their sole presence inside the neoplastic mass did not affect tumor growth.

We have also recorded the almost complete remission of the tumor in two mice immediately after the treatment, with mild signs of burn on the skin and more severe effects in the core portion of the tumor as a consequence of thermal energy release. Minor signs of tissue damage were visible also at the rim of the xenograft implant, while similar effects in the surrounding healthy tissues were completely absent. In a previous work [16], important results were reported in experimental breast tumors treated with MNs extracted from *M. magneticum* AMB-1 and exposed, three times in a week on alternate days, to an AMF of 183 kHz and 40 mT (∼32 kA/m) strength. Another group [24] described valuable outcomes with chemically synthesized magnetic nanoparticles on a xenograft breast cancer model, although using a different protocol for AMF: animals were exposed for 60 min at days 0 and 7, at 435 kHz and 15.4 kA/m strength. Magnetosomes extracted from *M. gryphiswaldense* MSR-1 are proven to induce mild tissue damage at a frequency of 187 kHz and magnetic field strength of 23 kA/m (H_0_ν = 4.3 ∗ 10^9^ Am^−1^·s^−1^) [17]. Here, we have improved the MFH protocol and measured a global reduction of tumor growth, convincing antitumoral activity with settings (frequency = 110 kHz, AMF strength = 23 kA/m, and H_0_ν = 2.5 ∗ 10^9^ Am^−1^·s^−1^) that are much closer to those employed in human studies [9]. Moreover, these settings deliver a product H_0_ν which is below the estimated tolerance threshold limit for tissue safety, as reported by Hergt et al. [35], for small exposed volumes: H_0_ν < 5 ∗ 10^9^ Am^−1^·s^−1^. Nevertheless, it is worth to mention that the value H_0_ν = 2.5 ∗ 10^9^ reported by Herg et al. is not a well-established maximum value but rather an arbitrarily chosen reference value.

There are some key aspects that, despite a certain degree of similarity, mark essential differences between these studies: the tumor model, the nanoparticles used for therapy, and the application of AMF, hence the comparison is obviously not straightforward. Regarding thermal efficiency, all the magnetic materials perform very well: SAR values lean towards synthetic nanoparticles, but both types of natural MNs exhibit excellent physical and thermal properties. Remarkable results regarding thermoablation of the tumor (thanks to elevated hyperthermic temperatures) and significant shrinking of tumors with a much moderate, thus highly effective, temperature increase were reported. However, AMF settings proposed in these studies could generate nonspecific tissue heating caused by induced eddy currents [35]. Here, we obtained a substantial, although not complete, thermoablation of tumors and statistically relevant slowdown of their growth while retaining biosafety-compliant AMF settings, a less radical approach that maintains its therapeutic effectiveness under clinical constraints for patient's safety.

It is interesting to note that, despite the good results attained immediately after the treatment, one week later we observed recovery in the growth rate: an additional finding in agreement with the abovementioned studies. It can be hypothesized that active cancer cells survived on the outer regions of the lesion, as confirmed by morphological MR imaging. T_2_w images of animals that better responded to therapy with the almost complete remission of the tumor showed indeed the presence of some residual malignant tissue. This progressive and unwanted proliferation of the tumor mass might be explained by the route of administration: although carefully fractionated, intratumoral injections could not ensure a uniform distribution of nanoparticles within the mass. Elevated interstitial pressure, combined with irregularities in the circulating blood flow, is among the main issues that prevent the redistribution of MNs and their subsequent uptake by cancer cells [36]. To obtain a homogeneous distribution of MNs within the tumor tissue, an MRI-guided intratumoral administration of MNs is advisable. For the whole duration of the experiment, we did not record any evidence of degeneration or alterations in the distribution of nanoparticles: the substantial parenchymal stability, which was noninvasively observed by longitudinal MR imaging and verified at a later stage ex vivo by histology, suggests that repeated hyperthermia treatments after single administration of magnetic material are feasible with evident beneficial effects for tumor regression. This paper has some limitations regarding the translational validity of the experimental model of glioblastoma used. First, a recent investigation of the origin of the used cell line, U87MG commercially available, performed by advanced techniques of genetic profiling and transcriptome analysis [37] showed that the DNA profile of commercial U87MG cells is different from that of the original cell line. Second, the growth pattern and microenvironment of subcutaneous tumors are very different from those of intracranial tumors potentially limiting the translational validity of the obtained results. However, the abovementioned investigation [37] concluded that the commercially available U87MG cell line is of “CNS origin and is likely to be a bona fide human glioblastoma cell line,” and moreover, subcutaneous models of brain tumors are widely applied in pharmaceutical research. Although the abovementioned issues must be taken into account, the present study has translational validity. However, in future investigations, we plan to apply the developed technique to the orthotopic model of brain tumors obtained from other well-characterized publicly available cell lines.

## 5. Conclusions

Synthetic magnetic nanoparticles have been widely considered for a long time as diagnostic and therapeutic agents in preclinical and clinical studies [38–41]. In this work, we have explored the theranostic potential of chains of magnetosomes, magnetic nanoparticles naturally produced by magnetotactic bacteria. We have shown that MFH based on magnetosomes lead to a clear slowdown of tumor growth with significant therapeutic effects. These results were achieved with a measured temperature increase much lower than values commonly reported in the literature, and the temperature increase strongly depends on the simultaneous application of both MNs and AMF. The sole presence of MNs inside the tumor mass does not elicit any cytotoxic response nor significant effects on tumor volumes. Improvement to the proposed protocol could include optimization of AMF parameters and exposure times to better exploit the heating power of magnetosomes. Moreover, we have applied MRI to monitor the distribution of MNs within the tumor tissue along the therapeutic treatment, and we have demonstrated that inhomogeneous distribution of MNs inside tumor tissue may strongly limit their therapeutic efficacy. The functionalization of MNs to target U87MG cancer cells would also be desirable: a highly tissue-specific delivery would improve the uptake of magnetic material and thus the clinical outcome of therapies.

## Data Availability

Electronic supplementary information with detailed experimental methods is available online or from the authors.

## Supplementary Materials

Supplementary Materials The supplementary material file reports details on the experimental procedures followed in the paper. Specifically, it reports experimental details on cancer cell culture (Information S1), investigation of MNs uptake in cancer cells (Information S2), transmission electron microscopy (TEM) of cells incubated with MNs (Information S3), magnetic resonance imaging (Information S4), and histology (Information S5).
