# Supplementary material for: Magnetosomes Extracted from Magnetospirillum gryphiswaldense as Theranostic Agents in an Experimental Model of Glioblastoma
Source: Contrast Media Mol Imaging. 2018 Jul 11;2018:2198703. doi: 10.1155/2018/2198703 (PMC6079595; doi:10.1155/2018/2198703)
Supplement: Supplementary Materials — The supplementary material file reports details on the experimental procedures followed in the paper. Specifically, it reports experimental details on cancer cell culture (Information S1), investigation of MNs uptake in cancer cells (Information S2), transmission electron microscopy (TEM) of cells incubated with MNs (Information S3), magnetic resonance imaging (Information S4), and histology (Information S5). [file 2198703.f1.pdf]

Supplementary Information

Magnetosomes extracted from Magnetospirillum gryphiswaldense as theranostic agents in an experimental model of glioblastoma

Silvia Mannucci[1*] , Stefano Tambalo[2*] , Giamaica Conti[1], Leonardo Ghin[3], Alessio Milanese[2], Anna Carboncino[1], Elena Nicolato[1], Maria Rosaria Marinozzi[1], Donatella Benati[1], Roberto Bassi[3], Pasquina Marzola[4], Andrea Sbarbati[1, 3]

[1]Department of Neuroscience, Biomedicine and Movement Sciences, University of Verona, Strada Le Grazie 8, I-37134, Verona, Italy; [2]Consorzio INSTM, Via G. Giusti 9, I-50121 Firenze, Italy; [3]Department of Biotechnology, University of Verona, Strada Le Grazie 15, I-37134 Verona, Italy; [4]Department of Computer Science, University of Verona, Strada Le Grazie 15, I-37134 Verona, Italy.

*These authors contributed equally to the paper

#present address: Center for Neuroscience and Cognitive Systems @UniTn, Istituto Italiano di Tecnologia, Corso Bettini 31, 38068 Rovereto, Italy;

Information S1: *Cancer cells culture*

U87MG cell line (purchased by ATCC Manassas, VA), was cultured in Eagle's Minimum Essential Medium (EMEM) with 10% of Fetal Bovine Serum (FBS), 1% of a mix of penicillin/streptomycin 1:1 and 1% of L-glutamine 200 mM, in 25 cm^2 plates and incubated at 37°C in humidified air with 5% CO_2. Medium and L-glutamine were purchased by Sigma-Aldrich (Italy),

while serum and antibiotic mix were acquired by GIBCO Life Technologies (USA). When at confluence, cells were treated with trypsin-EDTA 1% (GIBCO Life Technologies, USA), harvested and centrifuged at 1200 rpm for 5 min. The supernatant was discarded and cells pellet was resuspended in 1 ml of complete medium, placed in 75 cm^2 plates and incubated at 37°C and 5% of CO_2 until 80% confluence was detectable.

Information S2: *MNs-uptake in cancer cells*

Batches of $2X10^5$ cells were seeded in specific glasses multiwell for optical microscopy (Falcon BD, Italy) with 1 ml of growth medium. Cells were incubated at 37°C in humidified air with 5% CO_2 for 24 h. Different concentrations of MNs (1 mg/ml, 0.5 mg/ml, 0.2 mg/ml) were added to the wells and incubated for 6 h, 12 h and 24 h. At the selected time points, the growth medium was discarded and cells were washed with 1 ml of 1X sterile phosphate buffer saline (PBS, GIBCO, Life Technologies, USA). Cells were fixed with 1 ml of 4% buffered formalin (Bioptica, Italy) for 30 min at room temperature. Once formalin was discarded, samples were double stained with Prussian Blue and Nuclear Fast Red (Bioptica, Italy).

Information S3: *Transmission Electron Microscopy (TEM) of cells incubated with MNs*

Whole mount bacteria of different samples of MNs, extracted and purified, were fixed with glutharaldehyde 2% in Sorensen buffer pH 7.4 for 2 h, post-fixed in 1% osmium tetroxide in aqueous solution for 2 h, dehydrated in graded concentrations of acetone. At the end of dehydrating process, samples were positioned in a multi-well grid for electron microscopy and observed using Philips Morgagni TEM and equipped with a Megaview II camera for digital image acquisition.

U87MG cancer cells, plated on a 2.4 cm culture glass, were positioned on the bottom of 3.5 cm Petri dishes and incubated at 37°C in humidified air with 5% of CO_2 in 3 ml of growth medium. After 24h, the growth medium was then discarded and replaced with fresh medium added with MNs at different concentrations: 1 mg/ml, 0.5 mg/ml, 0.2 mg/ml. Cells were incubated as previously described for 6 h, 12 h and 24 h. At each time point, the growth medium was discarded and cells were washed with 1 ml of 1X sterile phosphate buffer saline (PBS, GIBCO, Life Technologies,

USA).Cells were fixed with glutaraldehyde 2% in Sorensen buffer pH 7.4 for 2 h, then post-fixed in 1% osmium tetroxide (OsO_4) in aqueous solution for 2 h, and finally dehydrated in graded concentrations of acetone. At the end of dehydrating process, glasses were stained with lead citrate.

Information S4: *Magnetic Resonance Imaging*

MRI was performed using a Bruker tomograph operating at 4.7 T, equipped with an actively shielded gradient insert (Bruker, Germany) having a maximum gradient strength of 40 G/cm. T_2 and T_2* weighted images were acquired with the following parameters: T_2 w RARE 3D sequence with TR=1200 ms, TE_{eff}=47.5 ms, FOV=5x2.5x2.5 cm^3, NEX=1, MTX=256/128/32, Slice Thickness=0.78 mm; T_2*weighted FLASH gradient echo sequence with TR=400 ms, TE=4.4ms, flip angle=10°, FOV=5x2.5 cm, NEX=2, MTX=256/128, NSLICES=8, Slice Thickness=2 mm.

Information S5: *Histology*

At the end of the last imaging session, tumors were excised, washed with PBS 0.1 M and fixed with 10% buffered formalin for 4 h. Afterwards, samples were dehydrated with increasing concentration gradient of alcohol from 70% to 100% and then with xylene for final processing. Tissues were embedded in paraffin and sections of 5 μm were obtained and dried at 37°C for 24 h. Section were then stained with Prussian blue and Nuclear Fast Red (Bioptica, Italy).
